# Self-Healing of SiC-Al_2_O_3_-B_4_C Ceramic Composites at Low Temperatures

**DOI:** 10.3390/ma15020652

**Published:** 2022-01-15

**Authors:** Baoguo Wang, Rong Tu, Yinglong Wei, Haopeng Cai

**Affiliations:** 1 School of Materials Science and Engineering, Wuhan University of Technology, Wuhan 430070, China; wangbg1996@163.com (B.W.); wyl120517@163.com (Y.W.); 2 State Key Laboratory of Advanced Technology for Materials Synthesis and Processing, Wuhan University of Technology, Wuhan 430070, China; turong@whut.edu.cn; 3 Institute of Advanced Materials Manufacturing Equipment and Technology, Wuhan University of Technology, Wuhan 430070, China

**Keywords:** self-healing, SiC-Al_2_O_3_-B_4_C, flexural strength, low temperatures

## Abstract

Self-healing ceramics have been researched at high temperatures, but few have been considered at lower temperatures. In this study, SiC-Al_2_O_3_-B_4_C ceramic composite was compacted by spark plasma sintering (SPS). A Vickers indentation was introduced, and the cracks were healed between 600 °C and 800 °C in air. Cracks could be healed completely in air above 700 °C. The ceramic composite had the best healing performance at 700 °C for 30 min, recovering flexural strength of up to 94.2% of the original. Good crack-healing ability would make this composite highly useful as it could heal defects and flaws autonomously in practical applications. The healing mechanism was also proposed to be the result of the oxidation of B_4_C.

## 1. Introduction

Ceramics have been widely used in some important and complex environments due to their high hardness and bending strength, etc.; however, their main shortcoming is their brittleness. Researchers have studied various toughening systems and mechanisms, such as whisker-reinforced and toughened materials [1], self-toughening materials [2], and particle-dispersion toughened materials [3]. However, in the process of material processing and application, it is inevitable that they will be damaged and produce a few micro-cracks, which would seriously degrade the performance of the ceramic composite, and finally cause catastrophic damage [4,5,6,7]. Inspired by the biological self-healing, the cracks are expected to also heal spontaneously before causing damage, recovering more than 90% of the undamaged strength of the matrix.

The self-healing mechanism of ceramic materials have been proposed generally to be of two types; one is diffusive healing [8], the other one is that some components react with the environmental medium to generate a glass sealant, forming a line of defense, blocking pores and cracks, and generating an intelligent response that actively adapts to the external environment [9,10]. The self-healing components must have a speedy reactivity with the environmental medium to cause a volume expansion with a certain fluidity. In order to cover the actual operating temperatures of the ceramic matrix composite (CMC), we need to explore agents in a range of healing temperatures. Nowadays, healing agents applied at moderate temperatures have achieved the most attention, such as SiC, Si_3_N_4_ and new ternary MAX family ceramics [11,12,13]. Nakatani et al. [14] prepared Si_3_N_4_/Y_2_O_3_, Si_3_N_4_/SiC/Y_2_O_3_ and Si_3_N_4_/Y_2_O_3_/Al_2_O_3_ ceramics and studied the effect of healing conditions on their oxidation behavior, and found good crack-healing ability. Due to the requirements of the high temperature applications, some high temperature healing agents are emerging, such as MoSi_2_ [15], HfSi_2_ [16], Y_2_SiO_5_ [17], ZrSiO_4_ [18]. Wang et al. [19] sprayed ZrSi_2_-MoSi_2_ on the SiC-coated C/C ceramic composite by using an ultrasonic plasma spraying technology, which showed good self-healing performance at 1450 °C. However, there is little research on the healing agent at low temperatures less than 800 °C. Bei et al. [20] examined crack healing using an Al_2_O_3_ composite loaded with the Ti_2_Al_0_._5_Sn_0_._5_C MAX phase repair filler. When cracks occurred in the matrix, the strength recovered by 107% at 700 °C for 48 h, and TiO_2_, SnO_2_ and α-Al_2_O_3_ was detected in the cracks. Yoshioka et al. [21] reported the application of TiC particles as a healing agent in alumina-based composites, presenting that the tensile strength of damaged samples was completely restored after annealing at 800 °C for 1 h. Boron and borides [22] could act as the healing agents at lower temperatures. B_4_C reacts with oxygen at high temperatures to generate B_2_O_3_, which has a melting temperature of 450 °C to be fluidic and a volume expansion rate of about 250% to fill up the cracks. In order to ensure that the oxidation product B_2_O_3_ plays a role in healing and that the inflow cracks are healed, the healing temperature must be in the range of 600 °C to 800 °C.

In order for ceramics to heal themselves in the actual application, it is very important to perform thermal oxidation at a low temperature within a short period. In this paper, the effect of the healing temperature and healing time of B_4_C with 15% mass fraction on the crack healing behavior in SiC/Al_3_O_2_ base ceramics was investigated. The healing behavior was studied on the micro-structure and macro-properties, and the healing mechanism was also explored.

## 2. Materials and Methods

### 2.1. Fabrication of the Ceramic Composite

The raw materials of SiC, Al_2_O_3_, B_4_C and glass powder (softening temperature 900 °C, to reduce the sintering temperature), 3 μm in average particle size and 99.9% in purity, was weighed at the mass ratio of 70%SiC-30%Al_2_O_3_:B_4_C:glass powder = 65%:15%:20%. The powders were mixed and ball-milled with a little ethanol for 6 h, and then dried at 90 °C in a vacuum for 10 h. The mixed powder was put into a graphite die 25 mm in diameter and compacted by spark plasma sintering in a vacuum at *T_s_* = 1350 °C with the heating rate of 100 °C/min under a pressure of 30 MPa for the soaking time of 10 min. The sintered sample was cut into rectangular strips with a precision cutting machine, and ground on both sides of the sample. The two sides were polished to a mirror finish to remove the scratches produced during the sample preparation and cutting process. The four edges also need be chamfered at 45° to reduce the residual stress on the surface of the specimen during processing. Finally, the size of the standard sample is 3 × 4 × 20 mm.

A Vickers hardness test was used to prefabricate the cracks in order to simulate the cracks on the surface of ceramic materials, and the length of the cracks was determined by controlling the load. The cracks in this experiment were prefabricated under a load of 98 N for 15 s. The position of the indentation is located at the center of the sample. The healing of the cracks is conducted for various times (*t_h_* = 0–300 min) and temperatures (*T_h_* = 600–800 °C) with a heating rate of 10 °C/min in air.

### 2.2. Characterization of the Ceramic Composite

The strength of the sample before and after healing was measured by three-point bending (Electronic universal testing machine, Instron 5966, Instron, Norwood, MA, USA) with the span of 20 mm at a loading rate of 0.5 mm/min. The phase composition of the ceramic composite was identified by X-ray diffraction (XRD, Empyrean, Malvern PANanalytical, Malvern, UK) using Cu Kα radiation. The surface morphology of the cracks was observed using a field emission scanning electron microscope (FE-SEM, JSM-7500F, JEOL, Akishima, Japan). Energy dispersive X-ray spectroscopy (EDS) analysis was conducted by using a spectrometer attached to this SEM. The chemical compositions of surface oxides were studied by X-ray Photoelectron Spectroscopy (XPS, Escalab 250Xi, Thermo Fisher Scientific, Waltham, MA, USA). The thermodynamic data for stoichiometric phase was calculated using the HSC Chemistry thermodynamic software.

## 3. Results and Discussion

### 3.1. Composite Microstructure

**Figure 1:**
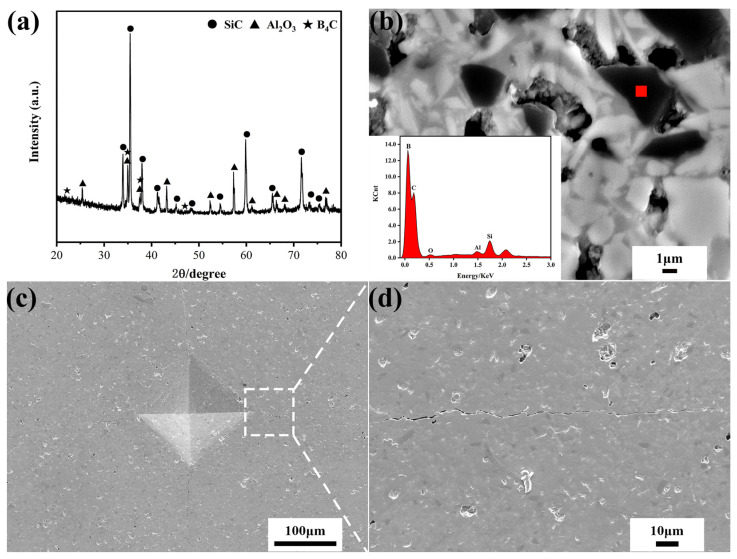
(**a**) X-ray diffraction pattern, back-scattering electron micrographs of phase composition (**b**) Vickers indentation (**c**) and crack (**d**) of SiC-Al_2_O_3_-B_4_C ceramic composite.

Figure 1a shows the XRD pattern of the sintered ceramic composites. The crystalline phases of SiC, Al_2_O_3_ only can be observed, indicating that there are no other side reactions during the high temperature sintering. Since the peak of B_4_C is very weak and overlaps with other peaks, it is difficult to be detected in XRD, whereas B_4_C was identified by EDS as shown in Figure 1b. At the dark region marked position, a large amount of B and C was detected. Indicating the dark phase is B_4_C. Meanwhile, the healing agent B_4_C is evenly dispersed in SiC/Al_2_O_3_ matrix, providing a prerequisite for cracks healing. Figure 1c,d shows the Vickers indentation and the magnified crack of ceramic composites. The pre-crack is to simulate the occurrence of cracks and facilitate the observation of crack healing during the study of self-healing performance.

### 3.2. Oxidation Induced Crack Healing

**Figure 2:**
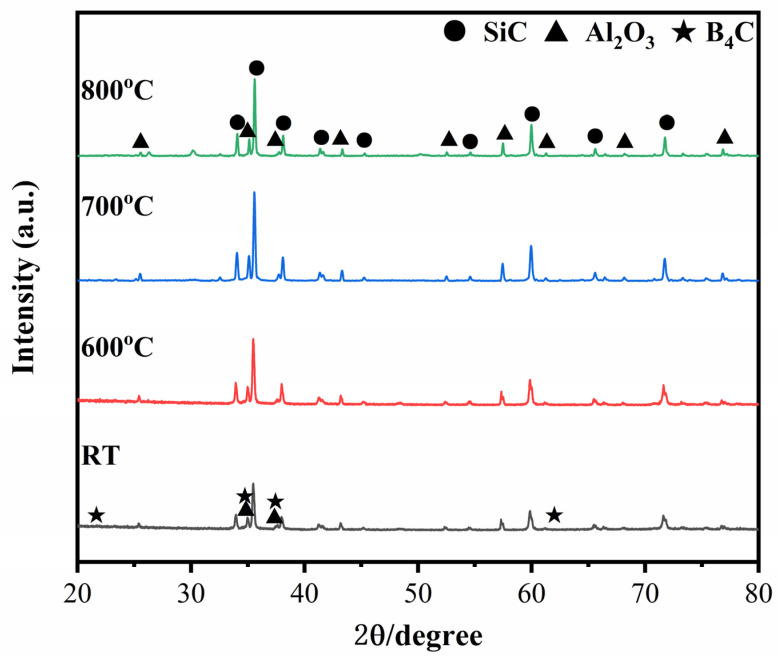
XRD patterns of ceramic composites healing for 60 min at *T_h_* = 25 °C, 600 °C, 700 °C and 800 °C.

Figure 2 presents the XRD patterns of the ceramic composite before and after healed at 600–800 °C for 60 min. After healing, the diffraction peak intensity of SiC and Al_2_O_3_ hardly changed at each temperature, suggesting that they did not participate in the oxidation process. The peak intensity of B_4_C was too weak to be detected out. In addition, the oxidation product of B_2_O_3_ was not detected in the XRD patterns due to the possible non-crystalline phase [23,24].

**Figure 3:**
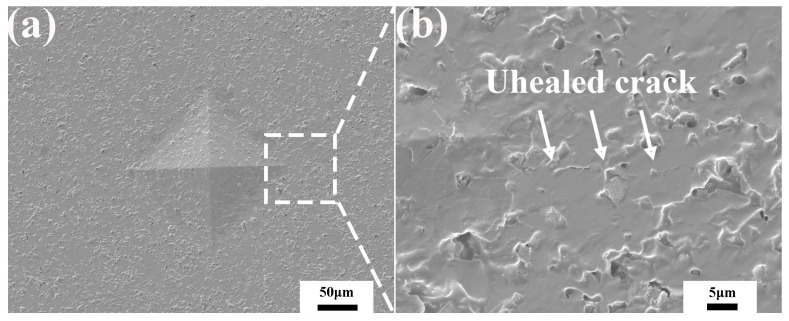

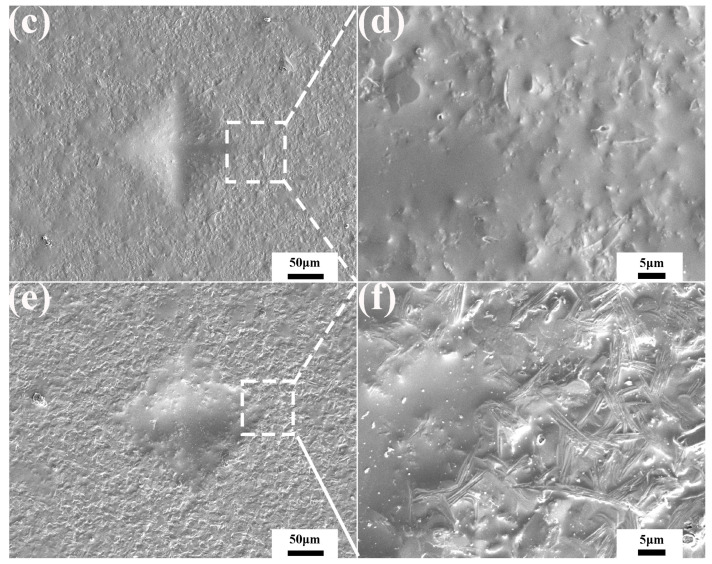
SEM morphology of Vickers indentation and cracks after healing at (**a**,**b**) 600 °C, (**c**,**d**) 700 °C and (**e**,**f**) 800 °C for 60 min.

Figure 3 shows the microstructure of the cracks healed at different temperatures for 60 min. After being annealed at 600 °C, as shown in Figure 3a, the length of the cracks decreased, indicating it was partially healed. The reason for partial healing was that the low oxidation rate of boron carbide at 600 °C could not produce enough oxide to fill the cracks. Although the cracks were not completely filled, it blunted the crack tip and prevented further propagation [25]. With increasing the healing temperature to 700 °C and 800 °C, the cracks disappeared completely and merged with the matrix.

**Figure 4:**
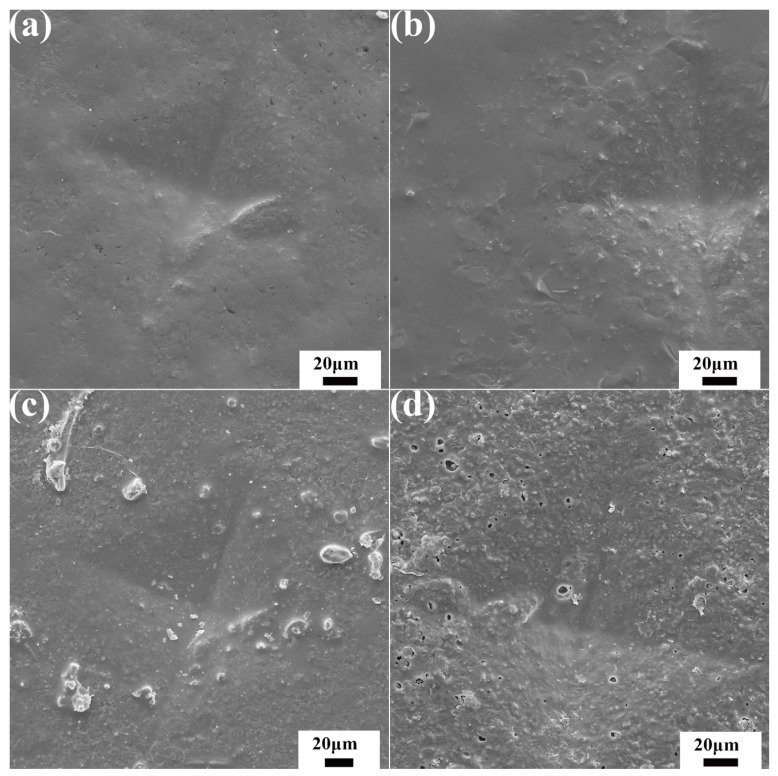
SEM images of Vickers indentation of ceramic materials after healing at 700 °C for (**a**) 30 min, (**b**) 90 min, (**c**) 180 min and (**d**) 300 min.

Figure 4 displays the morphology of Vickers indentation and cracks annealed at 700 °C for different time. The cracks on the diagonal of the indentation disappeared completely and the morphology around the indentation was relatively compact after healing for 30 min. On the other hand, many pores were formed at the indentation with the healing time of 300 min. Excessive healing time resulted in numerous pores and more defects, which seriously affected the properties of ceramic materials.

### 3.3. Recovery of Flexural Strength

**Figure 5:**
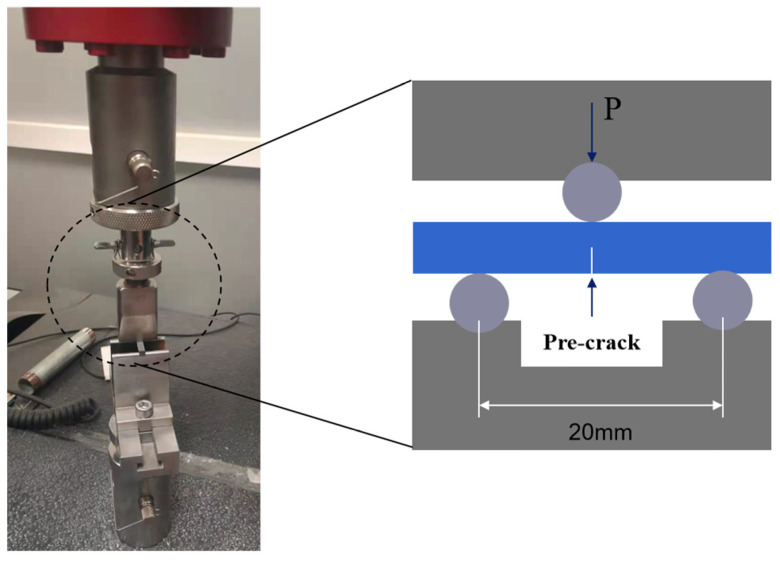
Schematic diagram of three-point bending of sample.

**Figure 6:**
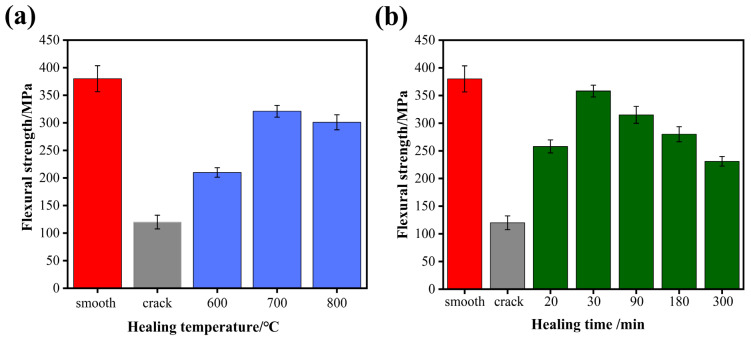
The flexural strength recovery of cracked specimens under different (**a**) healing temperature for 60 min, and; (**b**) healing time at 700 °C.

In order to evaluate the healing effect of the ceramic composite, a three-point bending test was carried out; the schematic diagram is shown in Figure 5. The flexural strength of the initial specimen and that with cracks were 380 ± 23.5 MPa and 120 ± 12.5 MPa, suggesting that the introduction of micro-cracks caused great damage to the mechanical properties. The flexural strength of the pre-crack specimens healed at different temperatures for 60 min, as shown in Figure 6a. After healed at 600 °C, the flexural strength increased to 200 ± 9.8 MPa, indicating a part of recovery. The specimens healed at 700 °C and 800 °C had the flexural strength of 321 ± 10.7 MPa and 301 ± 13.6 MPa, i.e., 84.5% and 79.2% of the initial flexural strength. At 800 °C, the decrease in flexural strength is due to the increase in oxidation rate, which leads to the increase in the oxidation degree of the material surface. Thus, the healing temperature was selected as 700 °C to investigate the effect of healing time on flexural strength, as shown in Figure 6b. The sample had the highest flexural strength of 358 ± 11.8 MPa, i.e., recovering up to 94.2% of the initial strength, after healed for 30 min. In practical applications, the material can respond quickly to heat after being damaged to avoid material failure. However, with increasing healing time, the recovery of the flexural strength weakened. The reason for the decrease in bending strength was that B_4_C was oxidized to produce a gaseous product CO_2_. Many micro-pores produced on the surface of the material became a new crack source when the gas pressure was large enough to escape.

**Figure 7:**
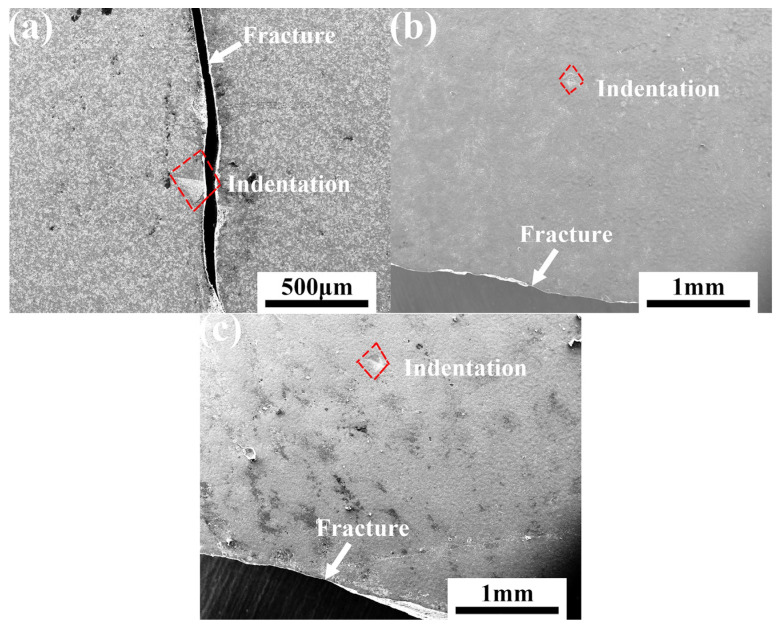
SEM image of the fracture path and indentation position of the sample after the bending test: the sample was healed for 60 min at (**a**) 600 °C, (**b**) 700 °C, (**c**) 800 °C.

Figure 7 displays the images of annealed specimens at different temperatures with the fracture path and indentation position, after the bending test of the composites. There were two kinds of fracture mode of ceramics composites. One mode identified that the fracture occurred across the indention point, as shown in Figure 7a, indicating that the oxidized product formed by healing agent was not enough to heal the crack. Meanwhile, the result was consistent with the low bend strength at 600 °C in Figure 6a. The other fracture mode initiated outside the pre-crack is shown in Figure 7 b,c, suggesting the crack had been repaired completely and the crack-healed region had the same or higher mechanical strength as the other regions.

### 3.4. Self-Healing Mechanism

**Figure 8:**
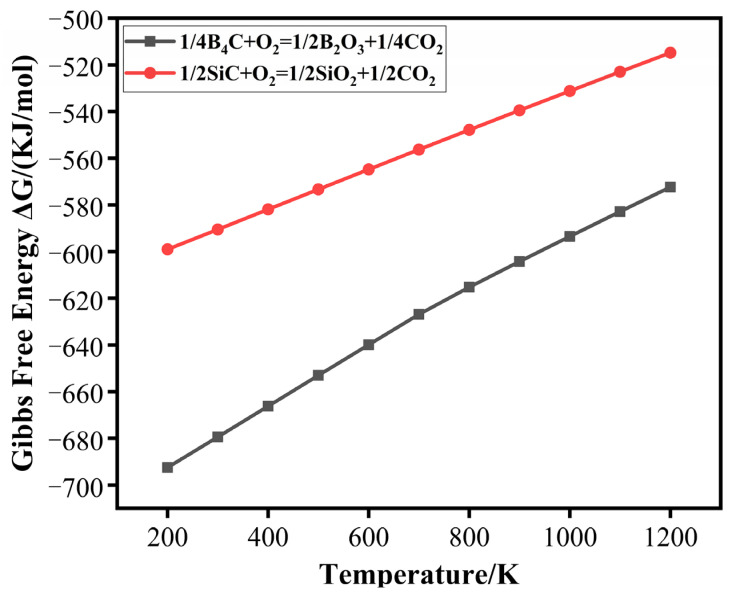
Standard Gibbs free energy of oxidation reaction of B_4_C and SiC.

Figure 8 shows the thermodynamic stability of B_4_C and SiC in air as a function of temperature. The Gibbs free energy (ΔG) of B_2_O_3_ and SiO_2_ is negative, which provides a theoretical bases to understand the appearances of B_2_O_3_ for healing and filling cracks. The Gibbs free energy of B_2_O_3_ is lower than SiO_2_, thus B_2_O_3_ should be generated preferentially. B_4_C can effectively provide self-healing behavior at 700–800 °C, whereas it has a low oxidation rate below 600 °C and is easy to form borate to volatilize above 900 °C [26]. The healing temperature was around 1200 °C once SiC was used as a healing agent alone [27]. Therefore, the oxidation behavior of B_4_C occurred mainly at 600–800 °C.

As crack healing was attributed to the oxidation reaction of the healing agent, the recovery of performance depended mainly on the healing time and temperature. The samples were observed before and after heat treatment at 700 °C in air; it was observed that the crack disappeared, and bend strength improved. For the weak diffraction peaks of the healing agent B_4_C and its oxidation product B_2_O_3_, as non-crystalline phases cannot be detected in the XRD patterns, the healing mechanism was not well explained.

**Figure 9:**
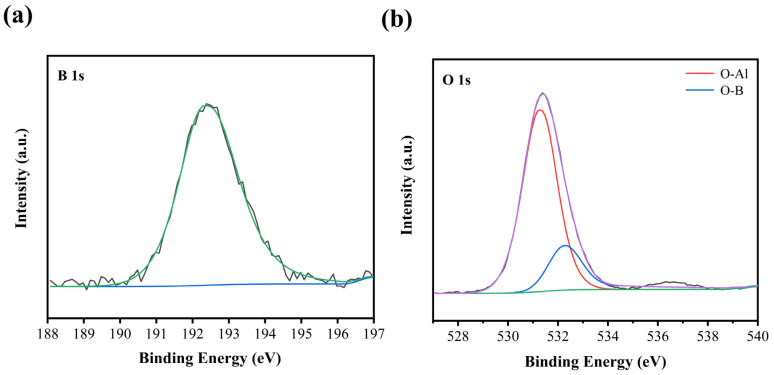
(**a**) B 1s, (**b**) O 1s XPS spectra of the specimen after heat treatment at 700 °C for 30 min.

In order to further explore the crack healing mechanism, the chemical composition of the oxidized surface was investigated by XPS. Figure 9 shows O 1s and B 1s spectra on the surface of ceramic composites after oxidation at 700 °C for 30 min, respectively. The B 1s spectrum with binding energy of 192.5 eV corresponds to B_2_O_3_ [28]. The O 1s spectrum had two peaks located at about 532.5 eV and 531.1 eV, which was assigned to B_2_O_3_ and Al_2_O_3_, respectively [29,30]. On account of alumina is the main component of matrix material, the oxidized product of the sample surface was B_2_O_3_ by the analysis of XPS, indicating that B_4_C participated in oxidation-induced crack healing.

**Figure 10:**
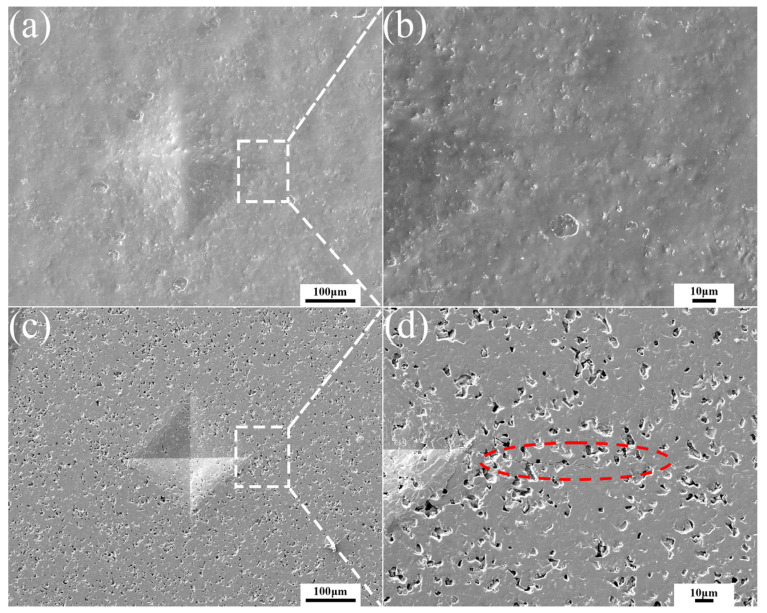
SEM image of Vickers indentation and cracks after heat treatment at 700 °C for 30 min in air, (**a**,**b**) with healing agent, (**c**,**d**) without healing agent.

**Figure 11:**
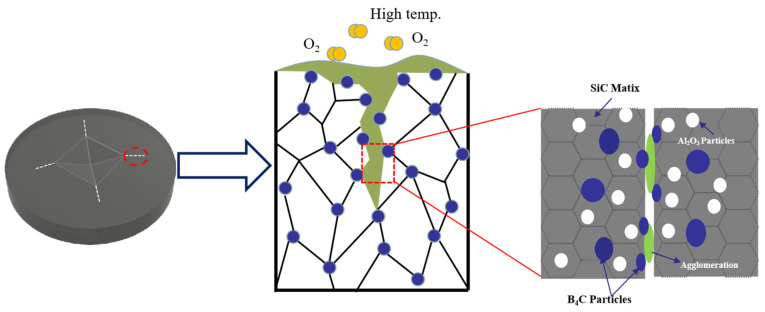
Healing mechanism diagram of SiC-Al_2_O_3_-B_4_C ceramic composite.

Figure 10 shows the SEM morphologies of the sample with and without healing agent B_4_C after heat treatment at 700 °C for 30 min in air. There were no signs of cracks around the indention observed in Figure 10a, indicating that the cracks healed completely. In contrast, the sample without healing agent, and after heat treatment, was not healed and numerous pores existed, as shown in Figure 10b. In conclusion, B_4_C as a healing agent had a good healing effect to repair the cracks; in addition to healing of the artificial indent cracks, closure of the residual porosity in the composite might also contribute to the strength recovery. Figure 11 shows a schematic of the mechanism crack healing at high temperatures. The healing agent around the crack was oxidized, resulting in oxide aggregation to fill the crack. It prevented further cracking and restored the mechanical properties of the matrix when the material was subjected to the force. Hence the reaction of crack healing to produce a new phase should be:

B_4_C + 4O_2_ = 2B_2_O_3_ + CO_2_ (1)

**Figure 12:**
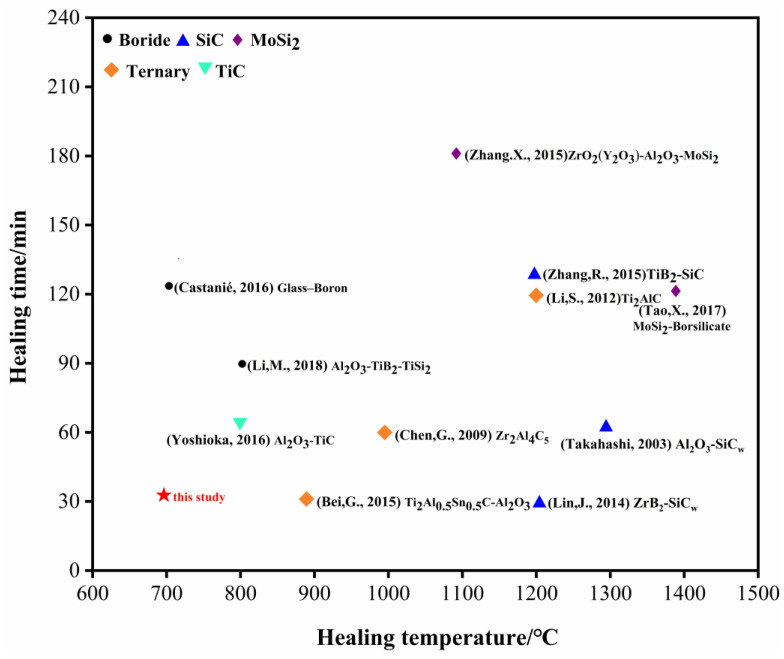
Time and temperature of crack healing in ceramic composites with different systems [9,12,21,22,27,31,32,33,34,35,36].

Figure 12 summarizes the time and temperature at which the crack is completely healed, as shown in the literature [9,12,21,22,27,31,32,33,34,35,36]. It can be seen that higher healing temperature or longer healing time is needed to achieve crack healing. The work of this paper realizes the rapid healing of prefabricated cracks at low temperatures, improves the mechanical properties of materials, and improves the service life and utilization rate of ceramic materials.

## 4. Conclusions

SiC-Al_2_O_3_-B_4_C self-healing ceramic composite was prepared by SPS technology, and the effects of healing temperature and healing time on the damaged SiC-Al_2_O_3_-B_4_C ceramic composite were studied. The crack was completely healed at 700 °C for 30 min, and the bending strength was restored to 94.2% of the original sample, i.e., 358 ± 11.8 MPa. The healing mechanism of SiC-Al_2_O_3_-B_4_C ceramic composite shows that B_2_O_3_, the oxidation product of B_4_C, flows into the cracks as liquid phase. After cooling, its volume expands to fill the cracks and recover the strength of SiC-Al_2_O_3_-B_4_C ceramic composite.

## Data Availability

The raw measured data of this study are available on request from the corresponding author.
